# Supplementary material for: Development of a novel transcriptomic measure of aging: Transcriptomic Mortality-risk Age (TraMA)
Source: Aging (Albany NY). 2025 Jun 13;17(6):1521–43. doi: 10.18632/aging.206272 (PMC12245202; doi:10.18632/aging.206272)
Supplement: Supplementary Figures [file aging-17-206272-s001.pdf]

SUPPLEMENTARY FIGURES

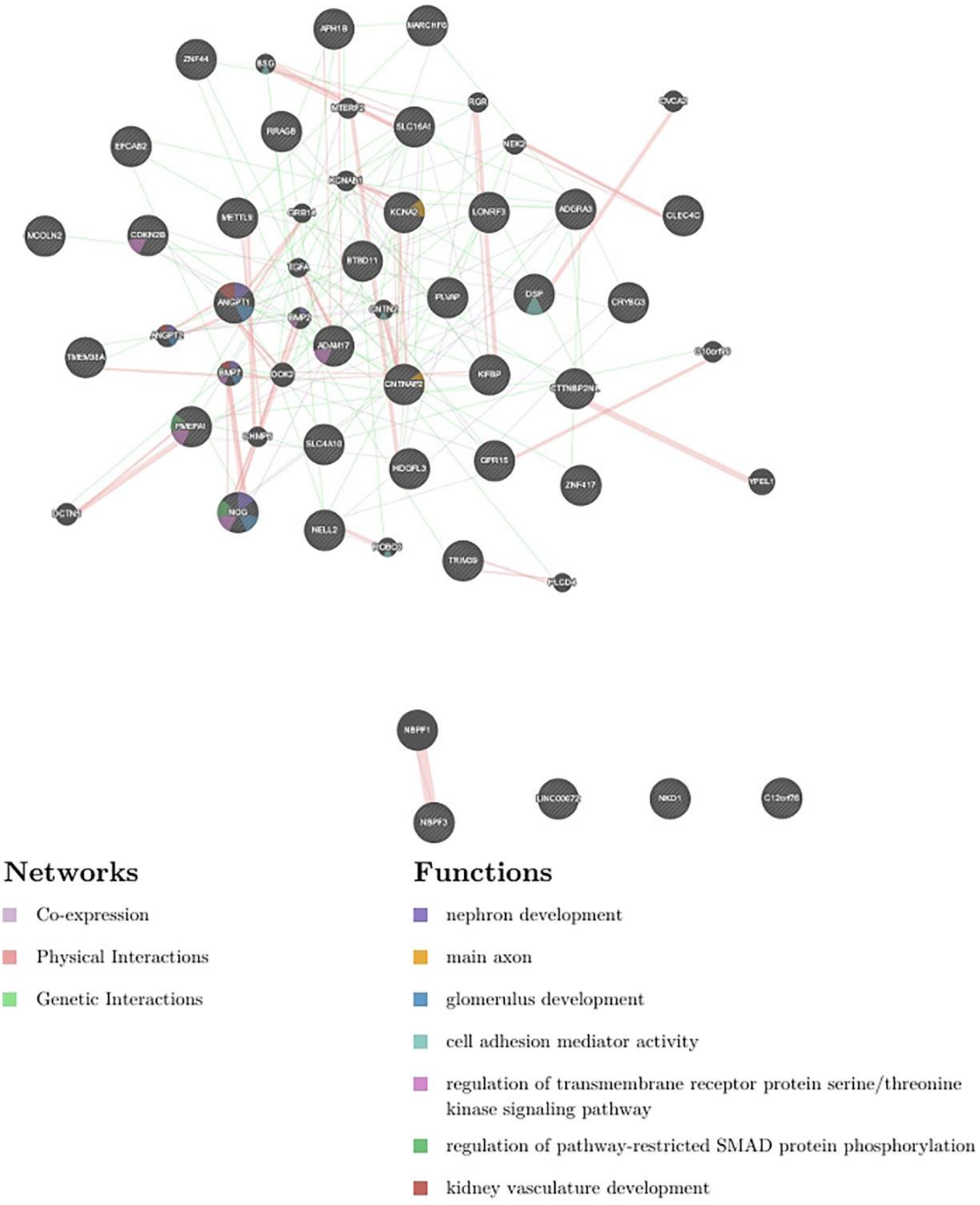

Supplementary Figure 1. Gene networks and functions from GeneMANIA analysis. Line color indicates type of network, and circle colors indicate functions of networks.

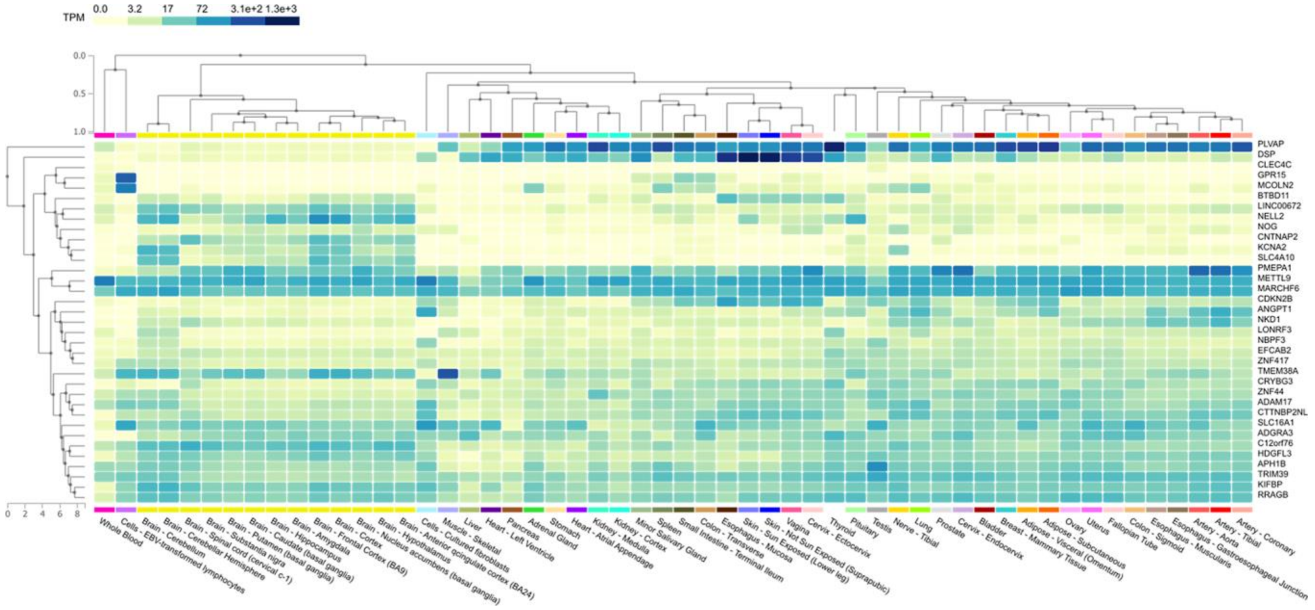

Supplementary Figure 2. Bulk expression levels of each TraMA gene from GTEx.

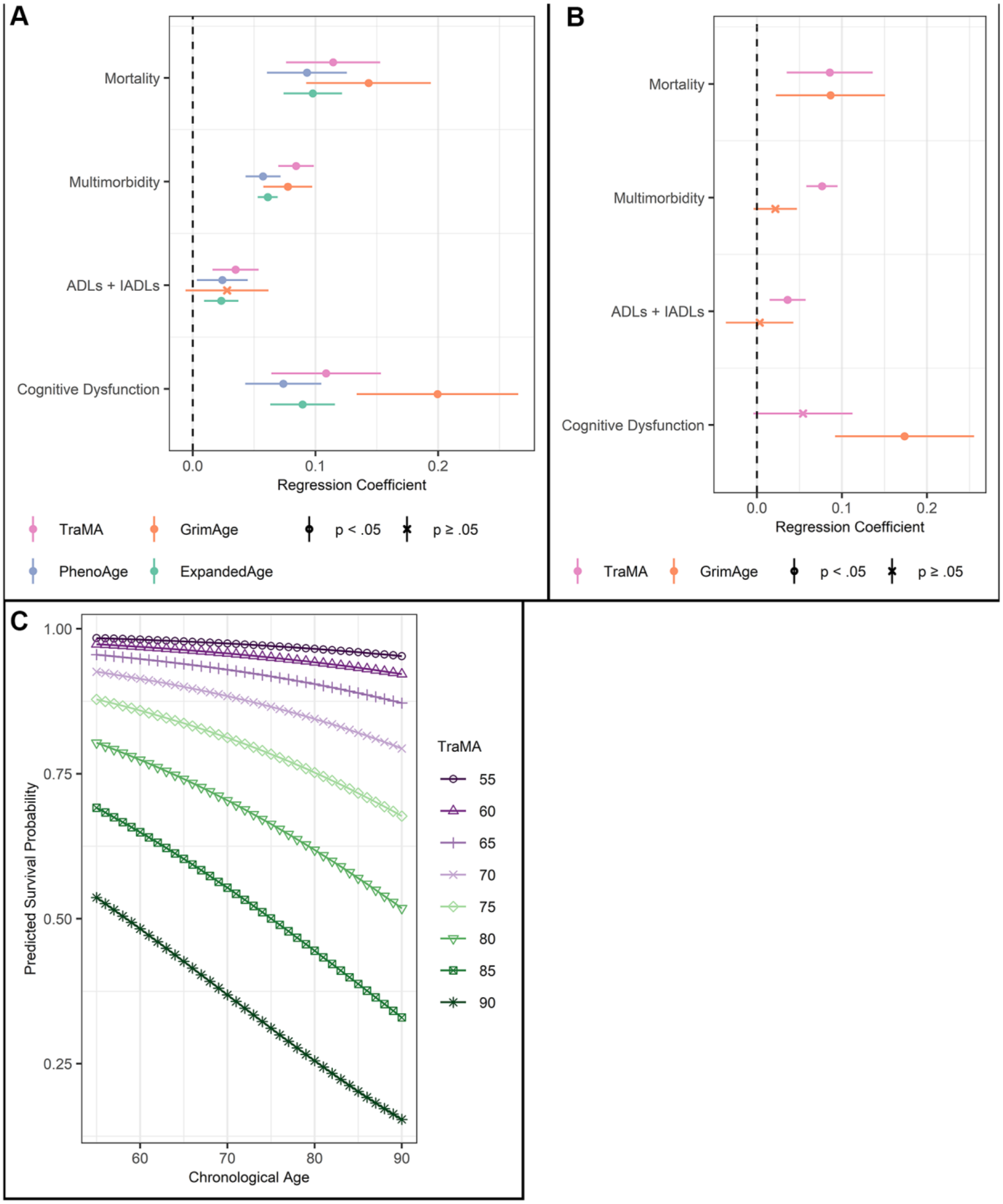

Supplementary Figure 3. (**A**) Regressions of health outcomes on biological aging measures; points represent regression coefficients and bars represent 95% confidence intervals; all models include age, race/ethnicity, sex/gender, and cell type as covariates; each point represents a separate regression equation. (**B**) Regressions of health outcomes on TraMA and GrimAge controlling for each other; points represent regression coefficients and bars represent 95% confidence intervals; all models include age, race/ethnicity, sex/gender, and cell type as covariates; each row represents a separate regression equation. (**C**) Predicted survival probabilities for imaginary White, not Hispanic male participants with mean levels of each cell type and varying ages and TraMA scores, using the same regression equation as shown in Figure 1D, Model 3 for HRS.

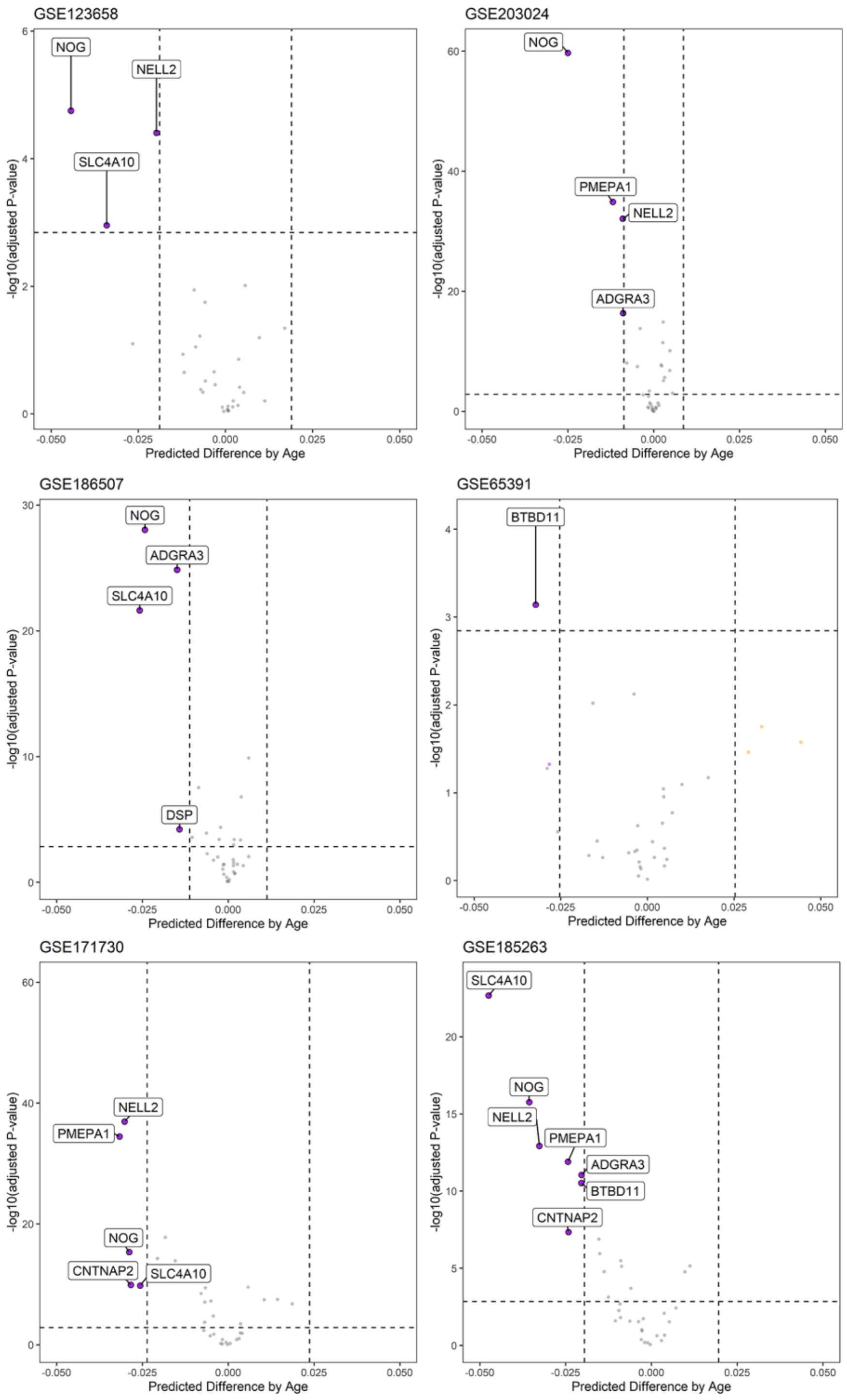

Supplementary Figure 4. Volcano plots from regressions of each gene in all of the human GEO datasets on age. NOG is significantly, negatively associated with age in all but 1 of these samples, suggesting it is robustly associated with aging. This is also one of the largest coefficients in the TraMA score.
