# Supplementary material for: Development of a novel transcriptomic measure of aging: Transcriptomic Mortality-risk Age (TraMA)
Source: Aging (Albany NY). 2025 Jun 13;17(6):1521–43. doi: 10.18632/aging.206272 (PMC12245202; doi:10.18632/aging.206272)
Supplement: Supplementary Tables 1 and 2 [file aging-17-206272-s002.pdf]

Supplementary Table 1. Genes in TraMA score and their ontologies.

Gene	GO term accession	GO term name	GO domain
	GO:0035640	exploration behavior	Biological Process
	GO:0050821	protein stabilization	Biological Process
	GO:0060395	SMAD protein signal transduction	Biological Process
	GO:0035249	synaptic transmission, glutamatergic	Biological Process
ABTB3	GO:0098978	glutamatergic synapse	Cellular Component
	GO:0016020	membrane	Cellular Component
	GO:0030165	PDZ domain binding	Molecular Function
	GO:0005515	protein binding	Molecular Function
	GO:0046982	protein heterodimerization activity	Molecular Function
	GO:0042987	amyloid precursor protein catabolic process	Biological Process
	GO:0030183	B cell differentiation	Biological Process
	GO:0007155	cell adhesion	Biological Process
	GO:0033627	cell adhesion mediated by integrin	Biological Process
	GO:0048870	cell motility	Biological Process
	GO:0071403	cellular response to high density lipoprotein particle stimulus	Biological Process
	GO:0071679	commissural neuron axon guidance	Biological Process
	GO:0050830	defense response to Gram-positive bacterium	Biological Process
	GO:0002467	germinal center formation	Biological Process
	GO:0006509	membrane protein ectodomain proteolysis	Biological Process
	GO:0120163	negative regulation of cold-induced thermogenesis	Biological Process
	GO:0030512	negative regulation of transforming growth factor beta receptor signaling pathway	Biological Process
	GO:0002446	neutrophil mediated immunity	Biological Process
	GO:0007220	Notch receptor processing	Biological Process
	GO:0007219	Notch signaling pathway	Biological Process
	GO:0043536	positive regulation of blood vessel endothelial cell migration	Biological Process
	GO:0030307	positive regulation of cell growth	Biological Process
	GO:0030335	positive regulation of cell migration	Biological Process
	GO:0008284	positive regulation of cell population proliferation	Biological Process
	GO:0032722	positive regulation of chemokine production	Biological Process
	GO:0045737	positive regulation of cyclin-dependent protein serine/threonine kinase activity	Biological Process
	GO:0045742	positive regulation of epidermal growth factor receptor signaling pathway	Biological Process
	GO:0045741	positive regulation of epidermal growth factor-activated receptor activity	Biological Process
	GO:1900087	positive regulation of G1/S transition of mitotic cell cycle	Biological Process
	GO:0002690	positive regulation of leukocyte chemotaxis	Biological Process
	GO:0010820	positive regulation of T cell chemotaxis	Biological Process
	GO:1903265	positive regulation of tumor necrosis factor-mediated signaling pathway	Biological Process
	GO:1905564	positive regulation of vascular endothelial cell proliferation	Biological Process
	GO:0019538	protein metabolic process	Biological Process
	GO:0016485	protein processing	Biological Process
	GO:0006508	proteolysis	Biological Process
	GO:0051128	regulation of cellular component organization	Biological Process
	GO:0033025	regulation of mast cell apoptotic process	Biological Process
ADAM17	GO:0001666	response to hypoxia	Biological Process
	GO:0032496	response to lipopolysaccharide	Biological Process
	GO:0050896	response to stimulus	Biological Process
	GO:0009410	response to xenobiotic stimulus	Biological Process
	GO:0048536	spleen development	Biological Process
	GO:0033077	T cell differentiation in thymus	Biological Process
	GO:0035313	wound healing, spreading of epidermal cells	Biological Process
	GO:0015629	actin cytoskeleton	Cellular Component
	GO:0016324	apical plasma membrane	Cellular Component
	GO:0009986	cell surface	Cellular Component
	GO:0005911	cell-cell junction	Cellular Component
	GO:0005737	cytoplasm	Cellular Component
	GO:0005829	cytosol	Cellular Component
	GO:0005788	endoplasmic reticulum lumen	Cellular Component
	GO:0005925	focal adhesion	Cellular Component
	GO:0000139	Golgi membrane	Cellular Component
	GO:0016020	membrane	Cellular Component
	GO:0045121	membrane raft	Cellular Component
	GO:0005886	plasma membrane	Cellular Component
	GO:0032587	ruffle membrane	Cellular Component
	GO:0004175	endopeptidase activity	Molecular Function
	GO:0016787	hydrolase activity	Molecular Function
	GO:0005178	integrin binding	Molecular Function
	GO:0005138	interleukin-6 receptor binding	Molecular Function
	GO:0046872	metal ion binding	Molecular Function
	GO:0004222	metalloendopeptidase activity	Molecular Function
	GO:1902945	metalloendopeptidase activity involved in amyloid precursor protein catabolic process	Molecular Function
	GO:0008237	metallopeptidase activity	Molecular Function
	GO:0005112	Notch binding	Molecular Function
	GO:0030165	PDZ domain binding	Molecular Function
	GO:0008233	peptidase activity	Molecular Function
	GO:0005515	protein binding	Molecular Function
	GO:0017124	SH3 domain binding	Molecular Function
	GO:0043120	tumor necrosis factor binding	Molecular Function
	GO:0007166	cell surface receptor signaling pathway	Biological Process
	GO:0007186	G protein-coupled receptor signaling pathway	Biological Process
	GO:0007165	signal transduction	Biological Process
	GO:0009897	external side of plasma membrane	Cellular Component
ADGRA3	GO:0016020	membrane	Cellular Component
	GO:0005886	plasma membrane	Cellular Component
	GO:0004930	G protein-coupled receptor activity	Molecular Function
	GO:0005515	protein binding	Molecular Function
	GO:0004888	transmembrane signaling receptor activity	Molecular Function
	GO:0007171	activation of transmembrane receptor protein tyrosine kinase activity	Biological Process
	GO:0001525	angiogenesis	Biological Process
	GO:0030154	cell differentiation	Biological Process
	GO:0031589	cell-substrate adhesion	Biological Process
	GO:0072012	glomerulus vasculature development	Biological Process
	GO:0030097	hemopoiesis	Biological Process
	GO:0030210	heparin biosynthetic process	Biological Process
	GO:0001701	in utero embryonic development	Biological Process
	GO:0043066	negative regulation of apoptotic process	Biological Process
	GO:0007162	negative regulation of cell adhesion	Biological Process
	GO:0002719	negative regulation of cytokine production involved in immune response	Biological Process
	GO:2000352	negative regulation of endothelial cell apoptotic process	Biological Process
	GO:0043524	negative regulation of neuron apoptotic process	Biological Process
	GO:0042308	negative regulation of protein import into nucleus	Biological Process
	GO:0001933	negative regulation of protein phosphorylation	Biological Process
	GO:0043116	negative regulation of vascular permeability	Biological Process
	GO:0051402	neuron apoptotic process	Biological Process
	GO:0050918	positive chemotaxis	Biological Process
	GO:0043536	positive regulation of blood vessel endothelial cell migration	Biological Process
	GO:1905605	positive regulation of blood-brain barrier permeability	Biological Process
	GO:0045785	positive regulation of cell adhesion	Biological Process
	GO:0010595	positive regulation of endothelial cell migration	Biological Process

	GO:0070374	positive regulation of ERK1 and ERK2 cascade	Biological Process
	GO:0010628	positive regulation of gene expression	Biological Process
ANGPT1	GO:0033138	positive regulation of peptidyl-serine phosphorylation	Biological Process
	GO:0050731	positive regulation of peptidyl-tyrosine phosphorylation	Biological Process
	GO:0051897	positive regulation of protein kinase B signaling	Biological Process
	GO:0001934	positive regulation of protein phosphorylation	Biological Process
	GO:0031398	positive regulation of protein ubiquitination	Biological Process
	GO:0002092	positive regulation of receptor internalization	Biological Process
	GO:0034394	protein localization to cell surface	Biological Process
	GO:0043122	regulation of I-kappaB kinase/NF-kappaB signaling	Biological Process
	GO:2000446	regulation of macrophage migration inhibitory factor signaling pathway	Biological Process
	GO:0014842	regulation of skeletal muscle satellite cell proliferation	Biological Process
	GO:0032680	regulation of tumor necrosis factor production	Biological Process
	GO:0002040	sprouting angiogenesis	Biological Process
	GO:0048014	Tie signaling pathway	Biological Process
	GO:0007169	transmembrane receptor protein tyrosine kinase signaling pathway	Biological Process
	GO:0062023	collagen-containing extracellular matrix	Cellular Component
	GO:0070062	extracellular exosome	Cellular Component
	GO:0005576	extracellular region	Cellular Component
	GO:0005615	extracellular space	Cellular Component
	GO:0045121	membrane raft	Cellular Component
	GO:0005902	microvillus	Cellular Component
	GO:0005886	plasma membrane	Cellular Component
	GO:0042802	identical protein binding	Molecular Function
	GO:0005515	protein binding	Molecular Function
	GO:0030971	receptor tyrosine kinase binding	Molecular Function
	GO:0005102	signaling receptor binding	Molecular Function
	GO:0042987	amyloid precursor protein catabolic process	Biological Process
	GO:0034205	amyloid-beta formation	Biological Process
	GO:0031293	membrane protein intracellular domain proteolysis	Biological Process
	GO:0007220	Notch receptor processing	Biological Process
	GO:0007219	Notch signaling pathway	Biological Process
	GO:0043085	positive regulation of catalytic activity	Biological Process
	GO:0010950	positive regulation of endopeptidase activity	Biological Process
	GO:0016485	protein processing	Biological Process
	GO:0005783	endoplasmic reticulum	Cellular Component
APH1B	GO:0005789	endoplasmic reticulum membrane	Cellular Component
	GO:0010008	endosome membrane	Cellular Component
	GO:0070765	gamma-secretase complex	Cellular Component
	GO:0000139	Golgi membrane	Cellular Component
	GO:0016020	membrane	Cellular Component
	GO:0005886	plasma membrane	Cellular Component
	GO:0030133	transport vesicle	Cellular Component
	GO:0061133	endopeptidase activator activity	Molecular Function
	GO:0005515	protein binding	Molecular Function
	GO:0030674	protein-macromolecule adaptor activity	Molecular Function
C12orf76	GO:0016020	membrane	Cellular Component
	GO:0007049	cell cycle	Biological Process
	GO:0031668	cellular response to extracellular stimulus	Biological Process
	GO:0031670	cellular response to nutrient	Biological Process
	GO:0071560	cellular response to transforming growth factor beta stimulus	Biological Process
	GO:0090398	cellular senescence	Biological Process
	GO:0001889	liver development	Biological Process
	GO:0030219	megakaryocyte differentiation	Biological Process
	GO:1902807	negative regulation of cell cycle G1/S phase transition	Biological Process
	GO:0008285	negative regulation of cell population proliferation	Biological Process
	GO:0050680	negative regulation of epithelial cell proliferation	Biological Process
	GO:2000134	negative regulation of G1/S transition of mitotic cell cycle	Biological Process
	GO:0060253	negative regulation of glial cell proliferation	Biological Process
	GO:0042326	negative regulation of phosphorylation	Biological Process
CDKN2B	GO:0030858	positive regulation of epithelial cell differentiation	Biological Process
	GO:0030511	positive regulation of transforming growth factor beta receptor signaling pathway	Biological Process
	GO:0000079	regulation of cyclin-dependent protein serine/threonine kinase activity	Biological Process
	GO:0070316	regulation of G0 to G1 transition	Biological Process
	GO:2000045	regulation of G1/S transition of mitotic cell cycle	Biological Process
	GO:0034097	response to cytokine	Biological Process
	GO:0014070	response to organic cyclic compound	Biological Process
	GO:0048536	spleen development	Biological Process
	GO:0005737	cytoplasm	Cellular Component
	GO:0005829	cytosol	Cellular Component
	GO:0005634	nucleus	Cellular Component
	GO:0004861	cyclin-dependent protein serine/threonine kinase inhibitor activity	Molecular Function
	GO:0005515	protein binding	Molecular Function
	GO:0019901	protein kinase binding	Molecular Function
	GO:0002250	adaptive immune response	Biological Process
	GO:0061760	antifungal innate immune response	Biological Process
	GO:0002376	immune system process	Biological Process
	GO:0045087	innate immune response	Biological Process
	GO:0009897	external side of plasma membrane	Cellular Component
	GO:0101003	ficolin-1-rich granule membrane	Cellular Component
CLEC4C	GO:0016020	membrane	Cellular Component
	GO:0005886	plasma membrane	Cellular Component
	GO:0030667	secretory granule membrane	Cellular Component
	GO:0070821	tertiary granule membrane	Cellular Component
	GO:0030246	carbohydrate binding	Molecular Function
	GO:0046872	metal ion binding	Molecular Function
	GO:0005515	protein binding	Molecular Function
	GO:0030534	adult behavior	Biological Process
	GO:0007420	brain development	Biological Process
	GO:0007155	cell adhesion	Biological Process
	GO:0008283	cell population proliferation	Biological Process
	GO:0021987	cerebral cortex development	Biological Process
	GO:0045163	clustering of voltage-gated potassium channels	Biological Process
	GO:0007612	learning	Biological Process
	GO:0021761	limbic system development	Biological Process
	GO:0031175	neuron projection development	Biological Process
	GO:0048812	neuron projection morphogenesis	Biological Process
	GO:0008038	neuron recognition	Biological Process
	GO:1903598	positive regulation of gap junction assembly	Biological Process
	GO:0060134	prepulse inhibition	Biological Process
	GO:0071205	protein localization to juxtaparanode region of axon	Biological Process
	GO:0035176	social behavior	Biological Process
	GO:0001964	startle response	Biological Process
	GO:0021756	striatum development	Biological Process
	GO:0071109	superior temporal gyrus development	Biological Process
	GO:0021794	thalamus development	Biological Process
	GO:0019226	transmission of nerve impulse	Biological Process
CNTNAP2	GO:0042297	vocal learning	Biological Process
	GO:0071625	vocalization behavior	Biological Process

	GO:0070161	anchoring junction	Cellular Component
	GO:0030673	axolemma	Cellular Component
	GO:0030424	axon	Cellular Component
	GO:0042995	cell projection	Cellular Component
	GO:0009986	cell surface	Cellular Component
	GO:0030425	dendrite	Cellular Component
	GO:0005769	early endosome	Cellular Component
	GO:0005794	Golgi apparatus	Cellular Component
	GO:0044224	juxtaparanode region of axon	Cellular Component
	GO:0016020	membrane	Cellular Component
	GO:0043025	neuronal cell body	Cellular Component
	GO:0033010	paranodal junction	Cellular Component
	GO:0033270	paranode region of axon	Cellular Component
	GO:0043204	perikaryon	Cellular Component
	GO:0097060	synaptic membrane	Cellular Component
	GO:0008076	voltage-gated potassium channel complex	Cellular Component
	GO:0019899	enzyme binding	Molecular Function
	GO:0002020	protease binding	Molecular Function
	GO:0005515	protein binding	Molecular Function
	GO:0044325	transmembrane transporter binding	Molecular Function
CRYBG3	GO:0008150	Biological Process	Biological Process
	GO:0002088	lens development in camera-type eye	Biological Process
	GO:0007601	visual perception	Biological Process
	GO:0032991	protein-containing complex	Cellular Component
	GO:0030246	carbohydrate binding	Molecular Function
	GO:0051018	protein kinase A binding	Molecular Function
	GO:0005212	structural constituent of eye lens	Molecular Function
CTTNBP2NL	GO:0034763	negative regulation of transmembrane transport	Biological Process
	GO:0032410	negative regulation of transporter activity	Biological Process
	GO:0006470	protein dephosphorylation	Biological Process
	GO:0015629	actin cytoskeleton	Cellular Component
	GO:0042995	cell projection	Cellular Component
	GO:0005737	cytoplasm	Cellular Component
	GO:0005856	cytoskeleton	Cellular Component
	GO:0030027	lamellipodium	Cellular Component
	GO:0001725	stress fiber	Cellular Component
	GO:0005515	protein binding	Molecular Function
	GO:0051721	protein phosphatase 2A binding	Molecular Function
DSP	GO:0034332	adherens junction organization	Biological Process
	GO:0086073	bundle of His cell-Purkinje myocyte adhesion involved in cell communication	Biological Process
	GO:0098609	cell-cell adhesion	Biological Process
	GO:0002934	desmosome organization	Biological Process
	GO:0008544	epidermis development	Biological Process
	GO:0090136	epithelial cell-cell adhesion	Biological Process
	GO:0045104	intermediate filament cytoskeleton organization	Biological Process
	GO:0045109	intermediate filament organization	Biological Process
	GO:0030216	keratinocyte differentiation	Biological Process
	GO:0018149	peptide cross-linking	Biological Process
	GO:0150105	protein localization to cell-cell junction	Biological Process
	GO:0086091	regulation of heart rate by cardiac conduction	Biological Process
	GO:0098911	regulation of ventricular cardiac muscle cell action potential	Biological Process
	GO:0043588	skin development	Biological Process
	GO:0003223	ventricular compact myocardium morphogenesis	Biological Process
	GO:0042060	wound healing	Biological Process
	GO:0005912	adherens junction	Cellular Component
	GO:0070161	anchoring junction	Cellular Component
	GO:0016323	basolateral plasma membrane	Cellular Component
	GO:0005911	cell-cell junction	Cellular Component
	GO:0001533	cornified envelope	Cellular Component
	GO:0005737	cytoplasm	Cellular Component
	GO:0005856	cytoskeleton	Cellular Component
	GO:0030057	desmosome	Cellular Component
	GO:0070062	extracellular exosome	Cellular Component
	GO:0005916	fascia adherens	Cellular Component
	GO:0101003	ficolin-1-rich granule membrane	Cellular Component
	GO:0014704	intercalated disc	Cellular Component
	GO:0005882	intermediate filament	Cellular Component
	GO:0016020	membrane	Cellular Component
	GO:0005634	nucleus	Cellular Component
	GO:0005886	plasma membrane	Cellular Component
	GO:0086083	cell adhesive protein binding involved in bundle of His cell-Purkinje myocyte communication	Molecular Function
	GO:0005515	protein binding	Molecular Function
	GO:0005080	protein kinase C binding	Molecular Function
	GO:0003723	RNA binding	Molecular Function
	GO:0097110	scaffold protein binding	Molecular Function
	GO:0005200	structural constituent of cytoskeleton	Molecular Function
	GO:0005198	structural molecule activity	Molecular Function
EFCAB2	GO:0042995	cell projection	Cellular Component
	GO:0005929	cilium	Cellular Component
	GO:0005737	cytoplasm	Cellular Component
	GO:0005856	cytoskeleton	Cellular Component
	GO:0031514	motile cilium	Cellular Component
	GO:0097228	sperm principal piece	Cellular Component
	GO:0005509	calcium ion binding	Molecular Function
	GO:0005515	protein binding	Molecular Function
GPR15	GO:0001525	angiogenesis	Biological Process
	GO:0007186	G protein-coupled receptor signaling pathway	Biological Process
	GO:0007165	signal transduction	Biological Process
	GO:0072678	T cell migration	Biological Process
	GO:0046718	viral entry into host cell	Biological Process
	GO:0005737	cytoplasm	Cellular Component
	GO:0005768	endosome	Cellular Component
	GO:0016020	membrane	Cellular Component
	GO:0005886	plasma membrane	Cellular Component
	GO:0015026	coreceptor activity	Molecular Function
	GO:0004930	G protein-coupled receptor activity	Molecular Function
	GO:0005515	protein binding	Molecular Function
	GO:0001618	virus receptor activity	Molecular Function
HDGFL3	GO:0006338	chromatin remodeling	Biological Process
	GO:0046785	microtubule polymerization	Biological Process
	GO:0007026	negative regulation of microtubule depolymerization	Biological Process
	GO:0031175	neuron projection development	Biological Process
	GO:0007165	signal transduction	Biological Process
	GO:0005737	cytoplasm	Cellular Component
	GO:0005829	cytosol	Cellular Component
	GO:0005576	extracellular region	Cellular Component
	GO:0005654	nucleoplasm	Cellular Component
	GO:0005634	nucleus	Cellular Component
	GO:0008083	growth factor activity	Molecular Function

	GO:0008017	microtubule binding	Molecular Function
	GO:0015631	tubulin binding	Molecular Function
	GO:0021987	cerebral cortex development	Biological Process
	GO:0022038	corpus callosum development	Biological Process
	GO:0034220	monoatomic ion transmembrane transport	Biological Process
	GO:0006811	monoatomic ion transport	Biological Process
	GO:0019228	neuronal action potential	Biological Process
	GO:0021554	optic nerve development	Biological Process
	GO:0021633	optic nerve structural organization	Biological Process
	GO:0097623	potassium ion export across plasma membrane	Biological Process
	GO:0071805	potassium ion transmembrane transport	Biological Process
	GO:0006813	potassium ion transport	Biological Process
	GO:0051260	protein homooligomerization	Biological Process
	GO:0045188	regulation of circadian sleep/wake cycle, non-REM sleep	Biological Process
	GO:0014059	regulation of dopamine secretion	Biological Process
	GO:0060078	regulation of postsynaptic membrane potential	Biological Process
	GO:0099505	regulation of presynaptic membrane potential	Biological Process
	GO:0019233	sensory perception of pain	Biological Process
	GO:0055085	transmembrane transport	Biological Process
	GO:0070161	anchoring junction	Cellular Component
	GO:0030424	axon	Cellular Component
	GO:0043194	axon initial segment	Cellular Component
	GO:0043679	axon terminus	Cellular Component
	GO:0044305	calyx of Held	Cellular Component
	GO:0042995	cell projection	Cellular Component
	GO:0030425	dendrite	Cellular Component
	GO:0005783	endoplasmic reticulum	Cellular Component
KCNA2	GO:0005789	endoplasmic reticulum membrane	Cellular Component
	GO:0098978	glutamatergic synapse	Cellular Component
	GO:0034702	ion channel complex	Cellular Component
	GO:0044224	juxtaparanode region of axon	Cellular Component
	GO:0030027	lamellipodium	Cellular Component
	GO:0031258	lamellipodium membrane	Cellular Component
	GO:0016020	membrane	Cellular Component
	GO:0043005	neuron projection	Cellular Component
	GO:0032809	neuronal cell body membrane	Cellular Component
	GO:0033010	paranodal junction	Cellular Component
	GO:0043204	perikaryon	Cellular Component
	GO:0005886	plasma membrane	Cellular Component
	GO:0045211	postsynaptic membrane	Cellular Component
	GO:0034705	potassium channel complex	Cellular Component
	GO:0042734	presynaptic membrane	Cellular Component
	GO:0045202	synapse	Cellular Component
	GO:0097060	synaptic membrane	Cellular Component
	GO:0008076	voltage-gated potassium channel complex	Cellular Component
	GO:0005251	delayed rectifier potassium channel activity	Molecular Function
	GO:0019894	kinesin binding	Molecular Function
	GO:0005216	monoatomic ion channel activity	Molecular Function
	GO:0015271	outward rectifier potassium channel activity	Molecular Function
	GO:0005267	potassium channel activity	Molecular Function
	GO:0005515	protein binding	Molecular Function
	GO:1905030	voltage-gated monoatomic ion channel activity involved in regulation of postsynaptic membrane po	Molecular Function
	GO:0099508	voltage-gated monoatomic ion channel activity involved in regulation of presynaptic membrane pot	Molecular Function
	GO:0005249	voltage-gated potassium channel activity	Molecular Function
	GO:0030154	cell differentiation	Biological Process
	GO:0021952	central nervous system projection neuron axonogenesis	Biological Process
	GO:0001701	in utero embryonic development	Biological Process
	GO:0000226	microtubule cytoskeleton organization	Biological Process
	GO:0006839	mitochondrial transport	Biological Process
KIFBP	GO:0007399	nervous system development	Biological Process
	GO:1990535	neuron projection maintenance	Biological Process
	GO:0005737	cytoplasm	Cellular Component
	GO:0005856	cytoskeleton	Cellular Component
	GO:0005739	mitochondrion	Cellular Component
	GO:0019894	kinesin binding	Molecular Function
	GO:0005515	protein binding	Molecular Function
LASP1NB	GO:0016020	membrane	Cellular Component
	GO:0005737	cytoplasm	Cellular Component
LONRF3	GO:0046872	metal ion binding	Molecular Function
	GO:0005515	protein binding	Molecular Function
	GO:0061630	ubiquitin protein ligase activity	Molecular Function
	GO:0036503	ERAD pathway	Biological Process
	GO:0010498	proteasomal protein catabolic process	Biological Process
	GO:0043161	proteasome-mediated ubiquitin-dependent protein catabolic process	Biological Process
	GO:0070936	protein K48-linked ubiquitination	Biological Process
	GO:0016567	protein ubiquitination	Biological Process
	GO:0030433	ubiquitin-dependent ERAD pathway	Biological Process
	GO:0005783	endoplasmic reticulum	Cellular Component
	GO:0005789	endoplasmic reticulum membrane	Cellular Component
	GO:0000835	ER ubiquitin ligase complex	Cellular Component
MARCHF6	GO:0016020	membrane	Cellular Component
	GO:0019899	enzyme binding	Molecular Function
	GO:0046872	metal ion binding	Molecular Function
	GO:0005515	protein binding	Molecular Function
	GO:0016740	transferase activity	Molecular Function
	GO:0031624	ubiquitin conjugating enzyme binding	Molecular Function
	GO:0061630	ubiquitin protein ligase activity	Molecular Function
	GO:0004842	ubiquitin-protein transferase activity	Molecular Function
	GO:1990381	ubiquitin-specific protease binding	Molecular Function
	GO:0008270	zinc ion binding	Molecular Function
	GO:0002250	adaptive immune response	Biological Process
	GO:0070588	calcium ion transmembrane transport	Biological Process
	GO:0006816	calcium ion transport	Biological Process
	GO:0019722	calcium-mediated signaling	Biological Process
	GO:0002376	immune system process	Biological Process
	GO:0045087	innate immune response	Biological Process
	GO:1905517	macrophage migration	Biological Process
	GO:0098655	monoatomic cation transmembrane transport	Biological Process
	GO:0034220	monoatomic ion transmembrane transport	Biological Process
	GO:0006811	monoatomic ion transport	Biological Process
	GO:1990266	neutrophil migration	Biological Process
	GO:0071651	positive regulation of chemokine (C-C motif) ligand 5 production	Biological Process
	GO:2000343	positive regulation of chemokine (C-X-C motif) ligand 2 production	Biological Process
	GO:0032722	positive regulation of chemokine production	Biological Process
MCOLN2	GO:0071642	positive regulation of macrophage inflammatory protein 1 alpha production	Biological Process
	GO:0071639	positive regulation of monocyte chemotactic protein-1 production	Biological Process
	GO:0015031	protein transport	Biological Process
	GO:2000341	regulation of chemokine (C-X-C motif) ligand 2 production	Biological Process
	GO:0005768	endosome	Cellular Component

	GO:0031902	late endosome membrane	Cellular Component
	GO:0005764	lysosome	Cellular Component
	GO:0016020	membrane	Cellular Component
	GO:0005886	plasma membrane	Cellular Component
	GO:0055037	recycling endosome	Cellular Component
	GO:0055038	recycling endosome membrane	Cellular Component
	GO:0005262	calcium channel activity	Molecular Function
	GO:0042802	identical protein binding	Molecular Function
	GO:0005261	monoatomic cation channel activity	Molecular Function
	GO:0072345	NAADP-sensitive calcium-release channel activity	Molecular Function
METTL9	GO:0032259	methylation	Biological Process
	GO:0005783	endoplasmic reticulum	Cellular Component
	GO:0005739	mitochondrion	Cellular Component
	GO:0008168	methyltransferase activity	Molecular Function
	GO:0005515	protein binding	Molecular Function
	GO:0106370	protein-L-histidine N-pros-methyltransferase activity	Molecular Function
	GO:0016740	transferase activity	Molecular Function
NBPF3	GO:0005737	cytoplasm	Cellular Component
NELL2	GO:0071679	commissural neuron axon guidance	Biological Process
	GO:0009566	fertilization	Biological Process
	GO:0007399	nervous system development	Biological Process
	GO:0070050	neuron cellular homeostasis	Biological Process
	GO:0005737	cytoplasm	Cellular Component
	GO:0005576	extracellular region	Cellular Component
	GO:0005615	extracellular space	Cellular Component
	GO:0005509	calcium ion binding	Molecular Function
	GO:0008201	heparin binding	Molecular Function
	GO:0042802	identical protein binding	Molecular Function
	GO:0005515	protein binding	Molecular Function
	GO:0005080	protein kinase C binding	Molecular Function
NKD1	GO:0003401	axis elongation	Biological Process
	GO:0060026	convergent extension	Biological Process
	GO:0001754	eye photoreceptor cell differentiation	Biological Process
	GO:0090090	negative regulation of canonical Wnt signaling pathway	Biological Process
	GO:0030178	negative regulation of Wnt signaling pathway	Biological Process
	GO:0043410	positive regulation of MAPK cascade	Biological Process
	GO:2000052	positive regulation of non-canonical Wnt signaling pathway	Biological Process
	GO:0045732	positive regulation of protein catabolic process	Biological Process
	GO:2000096	positive regulation of Wnt signaling pathway, planar cell polarity pathway	Biological Process
	GO:0090249	regulation of cell migration involved in somitogenic axis elongation	Biological Process
	GO:0007525	somatic muscle development	Biological Process
	GO:0016055	Wnt signaling pathway	Biological Process
	GO:0005737	cytoplasm	Cellular Component
	GO:0016020	membrane	Cellular Component
	GO:0005886	plasma membrane	Cellular Component
	GO:0000159	protein phosphatase type 2A complex	Cellular Component
	GO:0005509	calcium ion binding	Molecular Function
	GO:0046872	metal ion binding	Molecular Function
	GO:0030165	PDZ domain binding	Molecular Function
	GO:0005515	protein binding	Molecular Function
NOG	GO:0048646	anatomical structure formation involved in morphogenesis	Biological Process
	GO:0055009	atrial cardiac muscle tissue morphogenesis	Biological Process
	GO:0048318	axial mesoderm development	Biological Process
	GO:0007411	axon guidance	Biological Process
	GO:0030509	BMP signaling pathway	Biological Process
	GO:0007420	brain development	Biological Process
	GO:0051216	cartilage development	Biological Process
	GO:0030154	cell differentiation	Biological Process
	GO:0021533	cell differentiation in hindbrain	Biological Process
	GO:0008283	cell population proliferation	Biological Process
	GO:0071773	cellular response to BMP stimulus	Biological Process
	GO:0007417	central nervous system development	Biological Process
	GO:1904888	cranial skeletal system development	Biological Process
	GO:0009953	dorsal/ventral pattern formation	Biological Process
	GO:0042733	embryonic digit morphogenesis	Biological Process
	GO:0060272	embryonic skeletal joint morphogenesis	Biological Process
	GO:0048706	embryonic skeletal system development	Biological Process
	GO:0003272	endocardial cushion formation	Biological Process
	GO:0007492	endoderm development	Biological Process
	GO:0001706	endoderm formation	Biological Process
	GO:0050673	epithelial cell proliferation	Biological Process
	GO:0001837	epithelial to mesenchymal transition	Biological Process
	GO:0035640	exploration behavior	Biological Process
	GO:0060325	face morphogenesis	Biological Process
	GO:0008543	fibroblast growth factor receptor signaling pathway	Biological Process
	GO:0030900	forebrain development	Biological Process
	GO:0061384	heart trabecula morphogenesis	Biological Process
	GO:0001701	in utero embryonic development	Biological Process
	GO:0060173	limb development	Biological Process
	GO:0060291	long-term synaptic potentiation	Biological Process
	GO:0060425	lung morphogenesis	Biological Process
	GO:0003149	membranous septum morphogenesis	Biological Process
	GO:0048762	mesenchymal cell differentiation	Biological Process
	GO:0001707	mesoderm formation	Biological Process
	GO:0042474	middle ear morphogenesis	Biological Process
	GO:0008045	motor neuron axon guidance	Biological Process
	GO:2001234	negative regulation of apoptotic signaling pathway	Biological Process
	GO:0048712	negative regulation of astrocyte differentiation	Biological Process
	GO:0030514	negative regulation of BMP signaling pathway	Biological Process
	GO:0090090	negative regulation of canonical Wnt signaling pathway	Biological Process
	GO:0062044	negative regulation of cardiac epithelial to mesenchymal transition	Biological Process
	GO:0060044	negative regulation of cardiac muscle cell proliferation	Biological Process
	GO:0061037	negative regulation of cartilage development	Biological Process
	GO:0045596	negative regulation of cell differentiation	Biological Process
	GO:0030336	negative regulation of cell migration	Biological Process
	GO:0060302	negative regulation of cytokine activity	Biological Process
	GO:0010629	negative regulation of gene expression	Biological Process
	GO:0045668	negative regulation of osteoblast differentiation	Biological Process
	GO:0060392	negative regulation of SMAD protein signal transduction	Biological Process
	GO:0000122	negative regulation of transcription by RNA polymerase II	Biological Process
	GO:0007399	nervous system development	Biological Process
	GO:0021999	neural plate anterior/posterior regionalization	Biological Process
	GO:0001839	neural plate morphogenesis	Biological Process
	GO:0001843	neural tube closure	Biological Process
	GO:0021915	neural tube development	Biological Process
	GO:0038092	nodal signaling pathway	Biological Process
	GO:0048570	notochord morphogenesis	Biological Process
	GO:0001649	osteoblast differentiation	Biological Process
	GO:0003151	outflow tract morphogenesis	Biological Process

	GO:0007389	pattern specification process	Biological Process
	GO:0061626	pharyngeal arch artery morphogenesis	Biological Process
	GO:0021983	pituitary gland development	Biological Process
	GO:0090190	positive regulation of branching involved in ureteric bud morphogenesis	Biological Process
	GO:0050679	positive regulation of epithelial cell proliferation	Biological Process
	GO:0010628	positive regulation of gene expression	Biological Process
	GO:0090193	positive regulation of glomerulus development	Biological Process
	GO:0045944	positive regulation of transcription by RNA polymerase II	Biological Process
	GO:0099171	presynaptic modulation of chemical synaptic transmission	Biological Process
	GO:0060513	prostatic bud formation	Biological Process
	GO:0030510	regulation of BMP signaling pathway	Biological Process
	GO:0040036	regulation of fibroblast growth factor receptor signaling pathway	Biological Process
	GO:0048168	regulation of neuronal synaptic plasticity	Biological Process
	GO:1990926	short-term synaptic potentiation	Biological Process
	GO:0001501	skeletal system development	Biological Process
	GO:0007224	smoothened signaling pathway	Biological Process
	GO:0035019	somatic stem cell population maintenance	Biological Process
	GO:0061053	somite development	Biological Process
	GO:0021510	spinal cord development	Biological Process
	GO:0048863	stem cell differentiation	Biological Process
	GO:0001657	ureteric bud development	Biological Process
	GO:0060676	ureteric bud formation	Biological Process
	GO:0001655	urogenital system development	Biological Process
	GO:0003223	ventricular compact myocardium morphogenesis	Biological Process
	GO:0060412	ventricular septum morphogenesis	Biological Process
	GO:0008542	visual learning	Biological Process
	GO:0042060	wound healing	Biological Process
	GO:0005576	extracellular region	Cellular Component
	GO:0005615	extracellular space	Cellular Component
	GO:0098793	presynapse	Cellular Component
	GO:0019955	cytokine binding	Molecular Function
	GO:0005515	protein binding	Molecular Function
	GO:0042803	protein homodimerization activity	Molecular Function
	GO:0032502	developmental process	Biological Process
	GO:0000165	MAPK cascade	Biological Process
	GO:0002693	positive regulation of cellular extravasation	Biological Process
	GO:0043114	regulation of vascular permeability	Biological Process
	GO:0033209	tumor necrosis factor-mediated signaling pathway	Biological Process
	GO:0005901	caveola	Cellular Component
PLVAP	GO:0009986	cell surface	Cellular Component
	GO:0005737	cytoplasm	Cellular Component
	GO:0070062	extracellular exosome	Cellular Component
	GO:0016020	membrane	Cellular Component
	GO:0048471	perinuclear region of cytoplasm	Cellular Component
	GO:0005886	plasma membrane	Cellular Component
	GO:0042802	identical protein binding	Molecular Function
	GO:0005515	protein binding	Molecular Function
	GO:0030521	androgen receptor signaling pathway	Biological Process
	GO:0009968	negative regulation of signal transduction	Biological Process
	GO:0060392	negative regulation of SMAD protein signal transduction	Biological Process
	GO:0030512	negative regulation of transforming growth factor beta receptor signaling pathway	Biological Process
	GO:0031901	early endosome membrane	Cellular Component
	GO:0005768	endosome	Cellular Component
	GO:0010008	endosome membrane	Cellular Component
PMEPA1	GO:0005794	Golgi apparatus	Cellular Component
	GO:0000139	Golgi membrane	Cellular Component
	GO:0043231	intracellular membrane-bounded organelle	Cellular Component
	GO:0016020	membrane	Cellular Component
	GO:0005886	plasma membrane	Cellular Component
	GO:0005515	protein binding	Molecular Function
	GO:0140311	protein sequestering activity	Molecular Function
	GO:0070412	R-SMAD binding	Molecular Function
	GO:0034198	cellular response to amino acid starvation	Biological Process
	GO:0071230	cellular response to amino acid stimulus	Biological Process
	GO:1990253	cellular response to leucine starvation	Biological Process
	GO:0009267	cellular response to starvation	Biological Process
	GO:0010507	negative regulation of autophagy	Biological Process
	GO:0032008	positive regulation of TOR signaling	Biological Process
	GO:1904263	positive regulation of TORC1 signaling	Biological Process
	GO:0008104	protein localization	Biological Process
	GO:0032006	regulation of TOR signaling	Biological Process
	GO:0005737	cytoplasm	Cellular Component
	GO:0005829	cytosol	Cellular Component
RRAGB	GO:1990131	Gtr1-Gtr2 GTPase complex	Cellular Component
	GO:0005765	lysosomal membrane	Cellular Component
	GO:0005764	lysosome	Cellular Component
	GO:0016020	membrane	Cellular Component
	GO:0005634	nucleus	Cellular Component
	GO:0005525	GTP binding	Molecular Function
	GO:0003924	GTPase activity	Molecular Function
	GO:0051020	GTPase binding	Molecular Function
	GO:0032561	guanyl ribonucleotide binding	Molecular Function
	GO:0016787	hydrolase activity	Molecular Function
	GO:0000166	nucleotide binding	Molecular Function
	GO:0005515	protein binding	Molecular Function
	GO:0051780	behavioral response to nutrient	Biological Process
	GO:1905039	carboxylic acid transmembrane transport	Biological Process
	GO:0071407	cellular response to organic cyclic compound	Biological Process
	GO:0007098	centrosome cycle	Biological Process
	GO:0042593	glucose homeostasis	Biological Process
	GO:0035873	lactate transmembrane transport	Biological Process
	GO:0006629	lipid metabolic process	Biological Process
	GO:0015728	mevalonate transport	Biological Process
	GO:0015718	monocarboxylic acid transport	Biological Process
	GO:0035879	plasma membrane lactate transport	Biological Process
	GO:1902600	proton transmembrane transport	Biological Process
	GO:0042867	pyruvate catabolic process	Biological Process
	GO:1901475	pyruvate transmembrane transport	Biological Process
	GO:0050796	regulation of insulin secretion	Biological Process
	GO:0032094	response to food	Biological Process
	GO:0071422	succinate transmembrane transport	Biological Process
	GO:0055085	transmembrane transport	Biological Process
	GO:0150104	transport across blood-brain barrier	Biological Process
	GO:0016324	apical plasma membrane	Cellular Component
	GO:0009925	basal plasma membrane	Cellular Component
SLC16A1	GO:0016323	basolateral plasma membrane	Cellular Component
	GO:0030054	cell junction	Cellular Component
	GO:0005813	centrosome	Cellular Component
	GO:0070062	extracellular exosome	Cellular Component

	GO:0043231	intracellular membrane-bounded organelle	Cellular Component
	GO:0016328	lateral plasma membrane	Cellular Component
	GO:0016020	membrane	Cellular Component
	GO:0005886	plasma membrane	Cellular Component
	GO:0045202	synapse	Cellular Component
	GO:0046943	carboxylic acid transmembrane transporter activity	Molecular Function
	GO:0042802	identical protein binding	Molecular Function
	GO:0015129	lactate transmembrane transporter activity	Molecular Function
	GO:0015650	lactate:proton symporter activity	Molecular Function
	GO:0015130	mevalonate transmembrane transporter activity	Molecular Function
	GO:0008028	monocarboxylic acid transmembrane transporter activity	Molecular Function
	GO:0097159	organic cyclic compound binding	Molecular Function
	GO:0005515	protein binding	Molecular Function
	GO:0015295	solute:proton symporter activity	Molecular Function
	GO:0015141	succinate transmembrane transporter activity	Molecular Function
	GO:0015293	symporter activity	Molecular Function
	GO:0022857	transmembrane transporter activity	Molecular Function
SLC4A10	GO:0015701	bicarbonate transport	Biological Process
	GO:0048854	brain morphogenesis	Biological Process
	GO:0006821	chloride transport	Biological Process
	GO:0015698	inorganic anion transport	Biological Process
	GO:0098660	inorganic ion transmembrane transport	Biological Process
	GO:0035641	locomotory exploration behavior	Biological Process
	GO:0098656	monoatomic anion transmembrane transport	Biological Process
	GO:0006820	monoatomic anion transport	Biological Process
	GO:0034220	monoatomic ion transmembrane transport	Biological Process
	GO:0006811	monoatomic ion transport	Biological Process
	GO:0035264	multicellular organism growth	Biological Process
	GO:0009791	post-embryonic development	Biological Process
	GO:1902600	proton transmembrane transport	Biological Process
	GO:0021860	pyramidal neuron development	Biological Process
	GO:0030641	regulation of cellular pH	Biological Process
	GO:0051453	regulation of intracellular pH	Biological Process
	GO:0006885	regulation of pH	Biological Process
	GO:0048172	regulation of short-term neuronal synaptic plasticity	Biological Process
	GO:0009416	response to light stimulus	Biological Process
	GO:0035725	sodium ion transmembrane transport	Biological Process
	GO:0006814	sodium ion transport	Biological Process
	GO:0055085	transmembrane transport	Biological Process
	GO:0007601	visual perception	Biological Process
	GO:0097440	apical dendrite	Cellular Component
	GO:0016324	apical plasma membrane	Cellular Component
	GO:0030424	axon	Cellular Component
	GO:0043679	axon terminus	Cellular Component
	GO:0097441	basal dendrite	Cellular Component
	GO:0016323	basolateral plasma membrane	Cellular Component
	GO:0097442	CA3 pyramidal cell dendrite	Cellular Component
	GO:0042995	cell projection	Cellular Component
	GO:0030425	dendrite	Cellular Component
	GO:0016328	lateral plasma membrane	Cellular Component
	GO:0016020	membrane	Cellular Component
	GO:0043025	neuronal cell body	Cellular Component
	GO:0043204	perikaryon	Cellular Component
	GO:0005886	plasma membrane	Cellular Component
	GO:0098794	postsynapse	Cellular Component
	GO:0098793	presynapse	Cellular Component
	GO:0036477	somatodendritic compartment	Cellular Component
	GO:0045202	synapse	Cellular Component
	GO:0022853	active monoatomic ion transmembrane transporter activity	Molecular Function
	GO:0015297	antiporter activity	Molecular Function
	GO:0015106	bicarbonate transmembrane transporter activity	Molecular Function
	GO:0008509	monoatomic anion transmembrane transporter activity	Molecular Function
	GO:0015291	secondary active transmembrane transporter activity	Molecular Function
	GO:0140892	sodium,bicarbonate:chloride antiporter activity	Molecular Function
	GO:0008510	sodium:bicarbonate symporter activity	Molecular Function
	GO:0005452	solute:inorganic anion antiporter activity	Molecular Function
	GO:0015293	symporter activity	Molecular Function
TMEM38A	GO:0071313	cellular response to caffeine	Biological Process
	GO:0007029	endoplasmic reticulum organization	Biological Process
	GO:0098662	inorganic cation transmembrane transport	Biological Process
	GO:0034220	monoatomic ion transmembrane transport	Biological Process
	GO:0006811	monoatomic ion transport	Biological Process
	GO:0071805	potassium ion transmembrane transport	Biological Process
	GO:0006813	potassium ion transport	Biological Process
	GO:0010881	regulation of cardiac muscle contraction by regulation of the release of sequestered calcium ion	Biological Process
	GO:0014808	release of sequestered calcium ion into cytosol by sarcoplasmic reticulum	Biological Process
	GO:0070062	extracellular exosome	Cellular Component
	GO:0016020	membrane	Cellular Component
	GO:0031965	nuclear membrane	Cellular Component
	GO:0005634	nucleus	Cellular Component
	GO:0016529	sarcoplasmic reticulum	Cellular Component
	GO:0033017	sarcoplasmic reticulum membrane	Cellular Component
	GO:0042802	identical protein binding	Molecular Function
	GO:0005267	potassium channel activity	Molecular Function
TRIM39	GO:0006915	apoptotic process	Biological Process
	GO:0007049	cell cycle	Biological Process
	GO:0007095	mitotic G2 DNA damage checkpoint signaling	Biological Process
	GO:0043124	negative regulation of I-kappaB kinase/NF-kappaB signaling	Biological Process
	GO:0032435	negative regulation of proteasomal ubiquitin-dependent protein catabolic process	Biological Process
	GO:2000059	negative regulation of ubiquitin-dependent protein catabolic process	Biological Process
	GO:2001235	positive regulation of apoptotic signaling pathway	Biological Process
	GO:0050821	protein stabilization	Biological Process
	GO:0016567	protein ubiquitination	Biological Process
	GO:1902806	regulation of cell cycle G1/S phase transition	Biological Process
	GO:0005737	cytoplasm	Cellular Component
	GO:0005829	cytosol	Cellular Component
	GO:0005739	mitochondrion	Cellular Component
	GO:0005634	nucleus	Cellular Component
	GO:0042802	identical protein binding	Molecular Function
	GO:0046872	metal ion binding	Molecular Function
	GO:0005515	protein binding	Molecular Function
	GO:0016740	transferase activity	Molecular Function
	GO:0061630	ubiquitin protein ligase activity	Molecular Function
	GO:0008270	zinc ion binding	Molecular Function
ZNF417	GO:0006355	regulation of DNA-templated transcription	Biological Process
	GO:0006357	regulation of transcription by RNA polymerase II	Biological Process
	GO:0005634	nucleus	Cellular Component
	GO:0003677	DNA binding	Molecular Function
	GO:0000981	DNA-binding transcription factor activity, RNA polymerase II-specific	Molecular Function

	GO:0046872	metal ion binding	Molecular Function
	GO:0005515	protein binding	Molecular Function
	GO:0000978	RNA polymerase II cis-regulatory region sequence-specific DNA binding	Molecular Function
ZNF44	GO:0006355	regulation of DNA-templated transcription	Biological Process
	GO:0006357	regulation of transcription by RNA polymerase II	Biological Process
	GO:0005634	nucleus	Cellular Component
	GO:0003677	DNA binding	Molecular Function
	GO:0000981	DNA-binding transcription factor activity, RNA polymerase II-specific	Molecular Function
	GO:0046872	metal ion binding	Molecular Function
	GO:0005515	protein binding	Molecular Function
	GO:0000977	RNA polymerase II transcription regulatory region sequence-specific DNA binding	Molecular Function

Supplementary Table 2. Genes in the TraMA score and their most associated traits from past.

Gene	Most Associated Traits (Number of Associations)
ZNF44	Body Height (4)<br>Base Metabolic Rate Measurement (1)<br>Migraine Disorder (1)
CRYBG3	
NOG	Body Height (9)<br>Lean Mass (9)<br>Body Fat Percentage (2)<br>Balding Measurement (1)<br>Base Metabolic Rate Measurement (1)
ABTB3	Body Mass Index (2)<br>Body Fat Percentage (1)<br>Body Height (1)
NELL2	Dental Caries (1)
ZNF417	Body Height (3)
CLEC4C	
PMEPA1	
TRIM39	Hypertension (9)<br>Rheumatoid Arthritis (9)<br>Multiple Sclerosis (8)<br>Systemic Lupus Erythematosis (7)<br>Psoriasis (6)
SLC4A10	Body Mass Index (2)<br>Body Weight (1)<br>Diastolic Blood Pressure (1)<br>Hypertension (1)<br>Systolic Blood Pressure (1)
CNTNAP2	
NKD1	Crohn's Disease (12)<br>Inflammatory Bowel Disease (7)<br>Body Height (3)
DSP	Body Height (3)<br>Pulse Rate (2)<br>Chronic Obstructive Pulmonary Disease (1)<br>Forced Expiratory Volume (1)<br>Idiopathic Pulmonary Fibrosis (1)
KIFBP	Body Height (6)<br>Lean Mass (5)<br>Schizophrenia (3)<br>Base Metabolic Rate Measurement (1)<br>Whole Body Water Mass (1)
ANGPT1	Intraocular Pressure Measurement (4)<br>Balding Measurement (1)<br>Body Height (1)<br>Body Mass Index (1)
ADGRA3	

PLVAP	Resting Heart Rate (9)<br>Lean Mass (5)<br>Pulse Rate (4)<br>Body Height (1)<br>Body Mass Index (1)
MCOLN2	
CTTNBP2NL	Hypertension (4)<br>Impedance Problem (4)<br>Systolic Blood Pressure (3)<br>Diastolic Blood Pressure (2)
LASP1NB	Asthma (2)<br>Body Height (1)<br>Forced Expiratory Volume (1)<br>Forced Expiratory Volume in 1 Second (1)<br>Low Density Lipoprotein (1)
SLC16A1	Diastolic Blood Pressure (8)<br>Systolic Blood Pressure (6)<br>Hypertension (2)<br>Rheumatoid Arthritis (2)<br>Birth Weight (1)
NBPF3	
KCNA2	
EFCAB2	Attention Deficit Hyperactivity Disorder (1)<br>Bone Mineral Density (1)
**CDKN2B**	Coronary Artery Disease (5)<br>Type 2 Diabetes Mellitus (3)<br>Angina (2)<br>Migraine Disorder (2)<br>Platelet Count (2)
TMEM38A	Body Height (2)<br>Lymphocyte Count (2)<br>Dental Caries (1)<br>Leukocyte Count (1)
C12orf76	Body Fat Percentage (3)<br>Schizophrenia (3)<br>Hypertension (2)<br>Base Metabolic Rate Measurement (1)<br>Body Weight (1)
HDGFL3	Impedance Problem (8)<br>Body Fat Percentage (6)<br>Body Height (4)<br>Resting Heart Rate (3)<br>Lean Mass (2)
RRAGB	
GPR15	
LONRF3	Fasting Blood Glucose Measurement (1)
MARCHF6	

**ADAM17**	Birth Weight (8)<br>Body Height (7)<br>Hip Circumference (3)<br>Age (1)<br>Body Mass Index (1)
APH1B	Alzheimer's Disease (5)<br>Pulse Rate (2)<br>Resting Heart Rate (2)<br>Schizophrenia (2)<br>Attention Deficit Hyperactivity Disorder (1)
**METTL9**	Age (1)<br>Schizophrenia (1)

Note: top associations from TWAS Atlas; bold genes were assocated with age.
