# Supplementary material for: Development of a novel transcriptomic measure of aging: Transcriptomic Mortality-risk Age (TraMA)
Source: Aging (Albany NY). 2025 Jun 13;17(6):1521–43. doi: 10.18632/aging.206272 (PMC12245202; doi:10.18632/aging.206272)
Supplement: Supplementary Tables 3 and 4 [file aging-17-206272-s003.pdf]

SUPPLEMENTARY TABLES

Supplementary Table 3. Gene ontology terms enriched among the list of genes identified in pathways and FDR corrected Q values from GeneMania analysis.

Function	FDR	Genes in network	Genes in genome
Nephron development	0.034	5	84
Main axon	0.034	4	38
Glomerulus development	0.034	4	42
Cell adhesion mediator activity	0.038	4	56
Regulation of transmembrane receptor protein serine/threonine kinase signaling pathway	0.038	6	201
Regulation of pathway-restricted SMAD protein phosphorylation	0.038	4	58
Kidney vasculature development	0.038	3	18
Transmembrane receptor protein serine/threonine kinase signaling pathway	0.038	7	282
Kidney development	0.038	5	115
Renal system vasculature development	0.038	3	18
Pathway-restricted SMAD protein phosphorylation	0.039	4	60
Regulation of epidermal growth factor-activated receptor activity	0.062	3	24
Endocardial cushion morphogenesis	0.065	3	25
Renal system development	0.081	5	151
Cardiac chamber morphogenesis	0.085	4	79
Urogenital system development	0.088	5	158
Neuron recognition	0.096	3	31

Supplementary Table 4. Correlations among aging measures in HRS.

	TraMA	PCGrimAge	PCPhenoAge	ExpandedAge	Age Accelerated TraMA	Age Accelerated PCGrimAge	Age Accelerated PCPhenoAge	Age Accelerated ExpandedAge
TraMA	–	0.835	0.838	0.744	0.657	0.329	0.397	0.283
PCGrimAge	0.835	–	0.840	0.719	0.279	0.479	0.273	0.152
PCPhenoAge	0.838	0.840	–	0.749	0.381	0.304	0.632	0.274
ExpandedAge	0.744	0.719	0.749	–	0.349	0.199	0.343	0.692
Age Accelerated TraMA	0.657	0.279	0.381	0.349	–	0.537	0.597	0.449
Age Accelerated PCGrimAge	0.329	0.479	0.304	0.199	0.537	–	0.520	0.303
Age Accelerated PCPhenoAge	0.397	0.273	0.632	0.343	0.597	0.520	–	0.443
Age Accelerated ExpandedAge	0.283	0.152	0.274	0.692	0.449	0.303	0.443	–

Age accelerated values are residuals from a regression of the aging measure on chronological age.
